# Clinical Utility of Whole RNA Sequencing for Fusion Detection in Acute Leukemia

**DOI:** 10.3390/cells15121048

**Published:** 2026-06-08

**Authors:** Namsoo Kim, Yu Jin Park, Young Kyu Min, Seoyoung Lim, Yu Jeong Choi, Seung-Tae Lee, Jong Rak Choi, Hongkyung Kim, Saeam Shin

**Affiliations:** 1 Department of Laboratory Medicine, Yonsei University College of Medicine, Severance Hospital, Seoul 03722, Republic of Korea; 2 Aerospace Medical Center, Republic of Korea Air Force, Cheongju 28187, Republic of Korea; 3 Department of Laboratory Medicine, Severance Hospital, 50-1 Yonsei-ro, Seodaemun-gu, Seoul 03722, Republic of Korea; 4 Department of Laboratory Medicine, Graduate School of Medical Science, Brain Korea 21 PLUS Project, Yonsei University College of Medicine, Seoul 03722, Republic of Korea; 5 Department of Laboratory Medicine, Kangbuk Samsung Hospital, Sungkyunkwan University School of Medicine, Seoul 03181, Republic of Korea; 6 Dxome Co., Ltd., Seongnam-si 13209, Republic of Korea; 7 Department of Laboratory Medicine, Chung-Ang University College of Medicine, Seoul 06794, Republic of Korea

**Keywords:** RNA sequencing, gene fusion, leukemia

## Abstract

Background: Gene fusions play a pivotal role in the pathogenesis and classification of hematologic malignancies. RNA sequencing (RNA-seq) has emerged as a powerful tool for detecting gene fusions; however, many clinical studies have focused on targeted RNA-seq, and optimal parameters for whole transcriptome RNA-seq remain uncertain. Methods: We retrospectively analyzed whole RNA-seq data from 301 patients diagnosed with acute leukemia between October 2022 and May 2025 to characterize the landscape of pathogenic gene fusions. Fusions were identified using the Arriba algorithm, and subsampling analyses were performed on cases with recurrent fusions to determine the minimum sequencing output required for reliable detection. Results: Pathogenic gene fusions were identified in 113 of 301 patients (37.5%). Whole RNA-seq detected fusions that were not identifiable by conventional assays, including *UBTF*::*ATXN7L3*, and highlighted frequent fusion events, such as *ZNF384* rearrangements. Subsampling analysis demonstrated that a sequencing output ≥ 100 million reads (moderate confidence) or ≥300 million reads (high confidence) was sufficient for 100% detection of recurrent fusions. Conclusions: Whole RNA-seq reliably detects clinically relevant gene fusions in acute leukemia, aligns well with conventional karyotyping results, and surpasses targeted RNA-seq in comprehensiveness. A sequencing output of at least 100 million reads is recommended for clinical fusion detection.

## 1. Introduction

A fusion gene is a hybrid gene formed by the union of two individual genes, and can arise from structural rearrangements such as translocations, inversions, deletions, transcriptional read-through of adjacent genes, or trans-splicing events [1,2,3]. These alterations can lead to the production of chimeric proteins with altered functions, potentially resulting in increased oncogene expression or reduced expression of tumor suppressor genes [4].

The fifth edition of the World Health Organization (WHO) classification [5,6] and the 2022 International Consensus Classification (ICC) [7,8] have recently revised the categorization of hematologic malignancies. A notable shift in these updates is the increasing emphasis on recurrent gene rearrangements and molecular markers as diagnostic criteria. For instance, certain genetic abnormalities now define disease entities with minimal consideration of the blast percentage, which was a key morphological criterion in previous classifications. This change highlights the growing importance of molecular diagnostics in the classification and diagnosis of hematologic neoplasms.

Next-generation sequencing (NGS) technologies have transformed our understanding of the human genome [9]. Among them, RNA sequencing (RNA-seq) has emerged as a powerful method for detecting gene fusions, as it enables transcriptome-wide analysis and captures a broad range of fusion transcripts that might be missed by DNA-based or targeted approaches [10,11].

However, accurate prediction of gene fusions from short-read RNA-seq data remains technically challenging due to the presence of various artifacts generated during library preparation and sequence alignment [12]. Although several studies have investigated the clinical utility of RNA-seq in hematologic malignancies, most have focused on targeted RNA-seq panels [13] or involved limited clinical datasets [1]. In particular, the optimal sequencing output (total number of reads) required to reliably detect gene fusions has not been studied systematically. Furthermore, there is a lack of consensus on the minimum number of split reads required to distinguish true fusion events from background noise in clinical samples [14,15,16]. In this retrospective study, we aim to characterize the distribution and landscape of pathogenic gene fusions identified through whole RNA-seq in real-world clinical samples of acute leukemia, and to propose an optimal read output for reliable detection of gene fusions using a subsampling approach.

## 2. Materials and Methods

### 2.1. Patients and Samples

We retrospectively analyzed patients diagnosed with acute leukemia at a tertiary care center between October 2022 and May 2025. Demographic and clinical information, including age, sex, and leukemia subtype, was collected from the medical records. The study was approved by the Institutional Review Board (IRB) of Severance Hospital, Seoul, Republic of Korea (IRB No. 4-2025-0464) on 10 June 2025. The requirement for informed consent was waived due to the retrospective design and the use of anonymized data.

### 2.2. RNA Extraction

Mononuclear cells and total RNA were isolated on the same day as specimens collected using Erythrocyte lysis buffer (Qiagen, Venlo, Netherlands; catalog no. 79217) and a QIAamp RNA Blood Mini Kit (Qiagen, catalog no. 52304) according to the manufacturer’s protocols. RNA quantity and quality were measured using a 4200 Tapestation (Agilent Technologies, Santa Clara, CA, USA) with RNA Screen Tape (Agilent, catalog no. 5067-5576). The RNA integrity number of total RNAs was greater than 7.0.

### 2.3. Library Preparation for Whole RNA-Seq

A TruSeq Stranded mRNA kit (Illumina, San Diego, CA, USA) was used to construct an mRNA-based library according to the user guide. In brief, 500 ng of total RNA was prepared by diluting to a volume of 50 µL with nuclease-free water. Then, the poly-A containing mRNA was selected and fragmented into small pieces. cDNA synthesis was conducted with SuperScriptTM IV VILOTM Master Mix (Invitrogen, catalog no. 11756050). The synthesized cDNA was subjected to the adenylate-3′-ends and adaptor ligation and amplified by PCR to generate the final library products, which were analyzed by the 4200 Tapestation (Agilent) with DNA100 Screen Tape (Agilent, catalog no. 5067-5582) to verify the size of the final product at ~260 bp.

### 2.4. Sequencing and Bioinformatic Analyses

The quantified final library products were used for cluster generation, and NGS was performed on the Illumina NovaSeq 6000 sequencer (Illumina) in 2 × 150 bp paired-end format according to the Illumina Paired-End Sequencing protocol. Data analysis was performed using an in-house algorithm for the TruSeq Stranded mRNA kit as follows: (1) The BCL files were converted into FASTQ format using the tool “bcl2fastq” by Illumina, and the products were demultiplexed and trimmed to remove index adapter sequences with Trimmomatic [17]. (2) Next, Arriba was applied to the FASTQ files to detect fusions in the data based on the default parameters [12]. (3) Reads were aligned to the human reference genome (GRCh37) using STAR [18], and (4) gene annotation was performed with Arriba and a customized pipeline. We identified a positive gene fusion event as a product with a high confidence score as called by Arriba and considered reciprocal fusion transcripts as a single fusion event. Split read count was determined by summing split reads 1 and 2 as calculated by Arriba. The in-frame status and relationships of the gene fusions were determined based on the results obtained from Arriba. The *UBTF*::*ATXN7L3* fusion was initially filtered out by Arriba as a read-through event. By default, Arriba enables the read-through filter to exclude fusion transcripts formed by transcriptional read-through between adjacent genes on the same strand, as these are typically considered artifacts rather than genuine gene rearrangements. *UBTF*::*ATXN7L3* is a known in-frame read-through spanning 11.3 kbp between *UBTF* exon 17/21 and a 5′ UTR splice site of *ATXN7L3*, and was removed by this default filtering step. To assess its presence, we reanalyzed the data with the read-through filter disabled. To evaluate the presence of *UBTF*::*ATXN7L3*, fusion-negative samples were additionally reanalyzed with Arriba’s default read-through filter disabled. Identified *UBTF*::*ATXN7L3* fusions were independently confirmed by Sanger sequencing.

### 2.5. Binary Alignment Map (BAM) Subsampling

To evaluate the robustness of fusion detection, subsampling analyses were performed on samples containing recurrent fusions in at least 2 cases. Based on the original datasets with >400 million reads, BAM files were subsampled to 12 levels: 3.125 M, 6.25 M, 12.5 M, 25 M, 50 M, 100 M, 150 M, 200 M, 250 M, 300 M, 350 M, and 400 M reads. For each subsampling condition, detection rates were assessed based on the presence of fusions at high-confidence only and medium-to-high confidence levels as reported by Arriba, based on algorithm considering medium- and high-confidence predictions only when accurately identifying recurrently fused genes to avoid inclusion of numerous false positives [12]. Additionally, the average number of split reads for each recurrent fusion was calculated per read depth. For visualization of gene fusions, Circos plots were generated using the circlize package [19] in R version 4.5.0.

### 2.6. Conventional Chromosome Karyotyping

To compare the result of whole RNA-seq, conventional chromosome karyotyping was conducted. Heparinized bone marrow aspirate was cultured, harvested, and analyzed following standard protocols for G-banding chromosome karyotyping according to the International System for Human Cytogenetic Nomenclature.

## 3. Results

### 3.1. Clinical Results of Whole RNA-Seq

A total of 301 patients was enrolled in the study, comprising 205 with acute myeloid leukemia (AML), 81 with B-lymphoblastic leukemia (B-ALL), 10 with T-lymphoblastic leukemia (T-ALL), and 5 with mixed phenotype acute leukemia (MPAL) (Table 1). Among these, pathogenic gene fusions were detected in 113 patients (37.5%), with varying frequencies across disease subtypes: AML (*n* = 60, 29.3%), B-ALL (*n* = 48, 59.3%), T-ALL (*n* = 2, 20.0%), and MPAL (*n* = 3, 60.0%). In addition, 18 fusion events of unknown clinical significance were identified, with a predominance in AML cases (*n* = 15).

**Figure 1:**
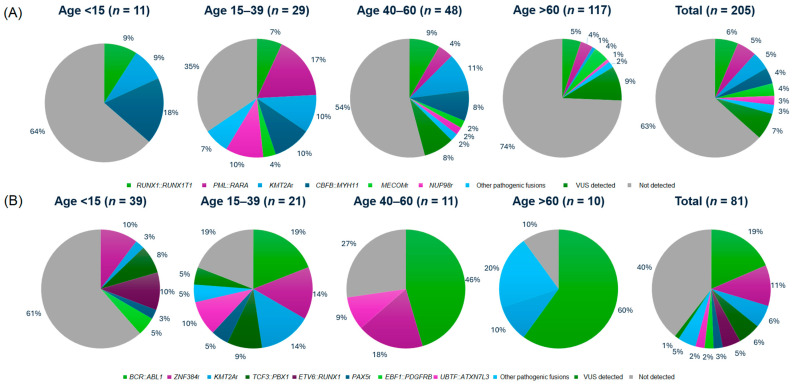
Age-stratified analysis of gene fusion detection in (**A**) acute myeloid leukemia and (**B**) B-lymphoblastic leukemia.

**Figure 2:**
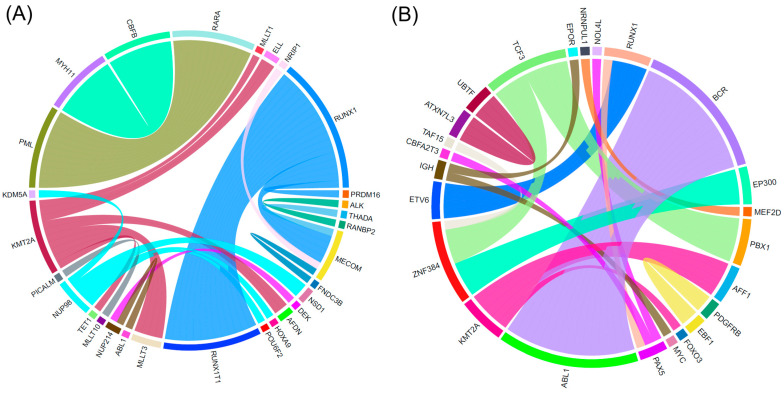
Circos plots of gene fusions detected by RNA sequencing. (**A**) Acute myeloid leukemia and (**B**) B-lymphoblastic leukemia.

We further analyzed the types of pathogenic fusions identified in these 113 patients. Age-stratified analysis was performed for AML and B-ALL, the two subtypes with sufficient sample sizes. Patients were grouped by age as follows: <15, 15–39, 40–60, and >60 years (Figure 1). In AML, the adolescent and young adult (AYA, 15–39 years) group showed a higher proportion of *PML*::*RARA* fusions than the other types, while the >60 year age group, which constituted most of AML patients, had the lowest frequency of rearrangements overall. In B-ALL, the prevalence of *BCR*::*ABL1* increased with age, with the <15 age group exhibiting the lowest frequency of rearrangements. Notably, rearrangements involving *ZNF384* were observed across all age groups ≤ 60 years, with a frequency ranging from 10% to 18%. To provide an intuitive visualization of these findings, two Circos plots were generated (Figure 2), highlighting rearrangements involving key genes such as *KMT2A*, *ZNF384*, and *NUP98*.

At our institution, orthogonal molecular assays are routinely performed as part of standard clinical workflows independent of whole RNA-seq results. RT-PCR assays for *BCR*::*ABL1* in B-ALL and *PML*::*RARA* in suspected acute promyelocytic leukemia are concurrently ordered to facilitate rapid therapeutic decision-making, including tyrosine kinase inhibitor or all-trans retinoic acid treatment initiation. In addition, *ETV6*::*RUNX1* FISH is routinely performed in pediatric B-ALL cases as part of cytogenetic evaluation. These orthogonal assays were therefore performed in a blinded manner with respect to RNA-seq findings. Complete concordance was observed between whole RNA-seq and orthogonal assays across all evaluated cases, including *BCR*::*ABL1* (15/81 B-ALL cases), *PML*::*RARA* (11/11 cases), and *ETV6*::*RUNX1* (4/44 pediatric B-ALL cases).

### 3.2. Detection of Fusions Not Covered by Targeted RNA-Seq Panels

We also examined fusions uniquely detectable by whole RNA-seq that would not be identified by other targeted fusion assays such as Archer FusionPlex Pan-Heme Panel (Invitae, San Francisco, CA, USA) or TruSight RNA Fusion Panel (Illumina, San Diego, CA, USA). This analysis was based on publicly available probe design and exon coverage information for each panel. Comparisons were not performed with HemaVision^®^ kit (DNA Technology, Aarhus, Denmark), which is limited to 28 target gene sites. Within the Archer FusionPlex Pan-Heme Panel, fusions not detectable due to probe design or exon coverage were: *EP300*::*ZNF384* (*n* = 4), *UBTF*::*ATXN7L3* (*n* = 3), *TAF15*::*ZNF384* (*n* = 1), *MEF2D*::*HNRNPUL1* (*n* = 1), *TCF3*::*ZNF384* (*n* = 1), *FNDC3B*::*MECOM* (*n* = 1), and *THADA*::*MECOM* (*n* = 1), the latter two involving non-targeted *MECOM* exons. Additionally, *IGH*::*MYC* (*n* = 1) was considered a caveat due to IGH complexity. Fusions undetectable by TruSight RNA Fusion Panel comprised *UBTF*::*ATXN7L3* (*n* = 3) and *MEF2D*::*HNRNPUL1* (*n* = 1). Reanalysis of fusion-negative samples with the read-through filter disabled additionally identified three *UBTF*::*ATXN7L3*-positive cases, all of which were independently confirmed by Sanger sequencing. Two female AYA B-ALL patients harbored *UBTF*::*ATXN7L3* fusions, consistent with prior reports. One patient experienced two relapses following allogeneic hematopoietic stem cell transplantation (allo-HSCT) and subsequently died 23 months post-diagnosis. Another was lost to follow-up after transfer, while the third remains scheduled for HSCT. Similarly, *MEF2D*::*HNRNPUL1* fusions are associated with aggressive clinical phenotypes [20]; a patient in our cohort developed two relapses post-allo-HSCT and is currently under life-sustaining treatment. This clinical trajectory aligns with established *UBTF*::*ATXN7L3* and *MEF2D*::*HNRNPUL1* alterations, both conferring poor prognosis in B-ALL.

### 3.3. Comparison with Conventional Chromosome Karyotyping

Last, we compared whole RNA-seq findings with conventional chromosome karyotyping results (Table 2). Among the 301 patients, two AML cases harbored cytogenetic abnormalities suggestive of *MECOM*- and *MYC*-associated rearrangements that were not detected by whole RNA-seq, likely because these abnormalities may not generate detectable fusion transcripts (Supplementary Table S1). Conversely, pathogenic fusions identified via whole RNA-seq were not observed by karyotyping in 32 patients (AML *n* = 9, B-ALL *n* = 19, T-ALL *n* = 2, MPAL *n* = 2). An additional 8 cases (AML *n* = 1, B-ALL *n* = 7) demonstrated successful fusion in whole RNA-seq but did not have available karyotyping results. There were *ZNF384* rearrangement in 9 cases, *ETV6*::*RUNX1* in 4 cases, and *BCR*::*ABL1* in 3 cases in B-ALL, and *NUP98* rearrangement in 3 cases and *KMT2A* rearrangement in 2 cases in AML. One B-ALL case demonstrated an additional rearrangement by whole RNA-seq that was missed by karyotyping.

### 3.4. Establishing the Optimal Sequencing Output for Clinical Whole RNA-Seq

To assess the minimum sequencing output (mapped reads) required for reliable fusion detection, we selected 52 samples containing 18 recurrent fusion types identified in leukemia and observed in at least 2 patients. Based on the original clinical datasets sequenced with >400 million reads, we performed subsampling at 12 levels: 3.125 M, 6.25 M, 12.5 M, 25 M, 50 M, 100 M, 150 M, 200 M, 250 M, 300 M, 350 M, and 400 M reads. Detection rates for each condition, based on Arriba results at high and medium/high confidence levels, are presented in Table 3.

At a threshold of 100 million reads, high-confidence detection was achieved in 94% of samples (49/52), with 3 cases missed (*ETV6*::*RUNX1* [*n* = 2], *NUP214*::*ABL1* [*n* = 1]). At ≥300 million reads, all fusions were detectable at high confidence. When a medium- or high-confidence criterion was applied, 94% of fusions (49/52) were detectable at 50 million reads, including *ETV6*::*RUNX1*, *NUP98*::*NSD1*, and *RUNX1*::*MECOM*. All fusions were detected at ≥100 million reads.

**Figure 3:**
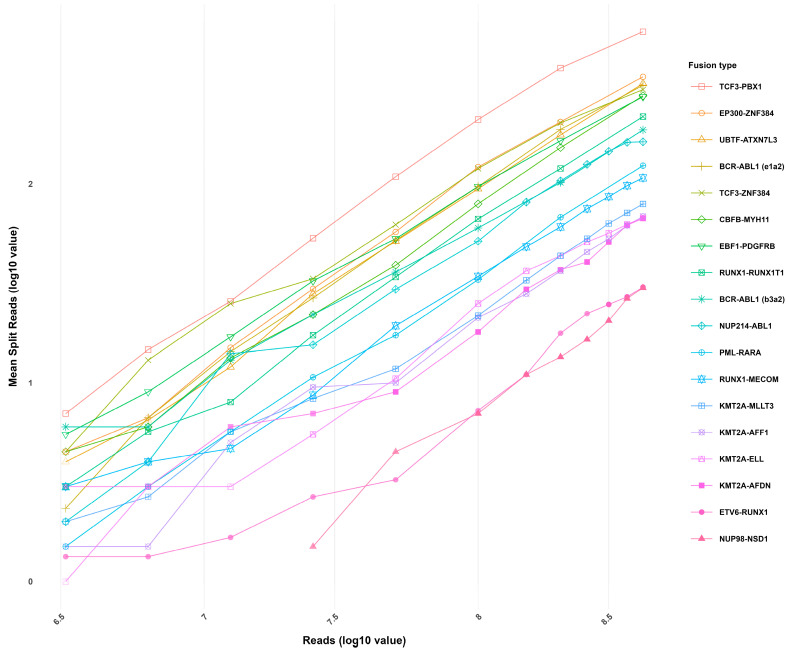
The mean number of split reads supporting each of the 18 fusion types at different reads in log scale.

The mean number of split reads supporting each of the 18 fusion types at different reads is shown in Figure 3. Consistent with the detection rates, fusions such as *ETV6*::*RUNX1* and *NUP98*::*NSD1* exhibited limited increases even with higher reads. A plateau in detection efficiency was generally observed beyond 300 million reads across multiple fusion types.

To methodologically validate our primary alignment-level benchmarks against potential sample-pool mapping biases, a representative subset of 14 cases underwent an independent validation pipeline using FASTQ-level downsampling via seqtk, followed by STAR alignment across five technical seeds per tier (Supplementary Figure S1). FASTQ level subsampling revealed 100% detection rate for moderate- or high-confidence calls at a minimum depth of 120 M reads, while high confidence was achieved for all variants at 450 M reads, excepting *NUP98*::*NSD1* at 80% recovery.

Profiling the background noise within this validation subset after excluding low-confidence calls and undetected reading frames revealed that false-positive artifacts paradoxically decreased at lower sequencing depths, possibly due to false-positive artifacts not passing Arriba’s scoring thresholds (Supplementary Table S3). Additionally, an exploratory downstream crosscheck against the Mitelman database effectively reduced these remaining spurious calls to near-zero across virtually all depth boundaries.

## 4. Discussion

In this study, we aimed to detect gene fusions that are difficult to identify with targeted RNA-seq using whole RNA-seq, and to establish real-world fusion statistics across a large cohort of acute leukemia patients. We also sought to determine the optimal sequencing output for accurate fusion detection using a subsampling approach.

Furthermore, our findings support the clinical applicability of whole RNA-seq not only as a complementary tool but as a potential replacement for conventional fusion assays such as fluorescence in situ hybridization(FISH)-based or targeted RNA-seq platforms. Whole RNA-seq enabled the detection of gene fusions across a diagnostic spectrum—including well-established fusions directly listed in the WHO 5th classification or ICC guidelines (e.g., *PML*::*RARA*) [5,6,7,8], emerging fusions with prognostic significance (e.g., *UBTF*::*ATXN7L3* and *MEF2D*::*HNRNPUL1*) [20,21], and rare fusions (e.g., *PARG*::*BMS1*) that may warrant further investigation in hematologic malignancies [22]. Importantly, at our institution, orthogonal validation—using reverse transcriptase polymerase chain reaction (RT-PCR) for *BCR*::*ABL1* and *PML*::*RARA*, and FISH for *ETV6*::*RUNX1*—showed complete concordance with RNA-seq results, even under blinded conditions, highlighting the high sensitivity and specificity of this method. Finally, through our subsampling approach, we demonstrated that pathogenic fusions could be robustly detected at read depths as low as 100 million reads, offering a practical and scalable solution for routine clinical laboratories with constraints in data processing or storage infrastructure.

Among the 301 patients, 40 who harbored pathogenic fusions presented normal or uninterpretable chromosomal karyotyping results—accounting for 10 of 205 AML cases, 26 of 81 B-ALL cases, 2 of 10 T-ALL cases, and 2 of 5 MPAL cases. The limited resolution of conventional G-banding explains some of these discrepancies, as it fails to detect cryptic rearrangements like *ETV6*::*RUNX1* and those involving *ZNF384* [23,24]. The 2 cases (0.7%) of known chromosomal abnormalities not detected by whole RNA-seq represent a marked improvement over the institution’s prior targeted RNA-seq results (4.5%, 12 of 264) [13]. The prior targeted RNA-seq cohort and the present whole RNA-seq cohort were derived from different clinical implementation periods at our institution, and overlapping transition-phase cases analyzed using both platforms were intentionally excluded from this study. This suggests that whole RNA-seq can better correlate with karyotyping and improve overall diagnostic concordance. Nevertheless, because certain subtypes—such as high hyperdiploidy or hypodiploidy in B-ALL—are better characterized by cytogenetics, and others (e.g., AML with *NPM1* mutation or B-ALL with *PAX5* alterations or *PAX5* p.80R) are more readily defined by DNA analysis, a comprehensive diagnostic approach integrating whole RNA-seq, DNA sequencing, and chromosomal analysis remains essential.

In addition to fusion detection, we analyzed the relationship between patient age and gene fusion pattern by categorizing cases of AML and B-ALL by age group. In AML, the overall detection rate of pathogenic fusions in this study was 29.3%, which was slightly lower than expected [25]. This was largely due to the relatively low detection rate of 16.2% in patients aged >60 years, who comprised 57.1% of the AML cohort. In contrast, the AYA (age 15–39 years) group demonstrated a notably high fusion detection rate of 65.5%, with *PML*::*RARA* and *NUP98* rearrangements particularly enriched (17.2% and 10.3%, respectively). In patients aged <15 years, only three fusion types were identified—*RUNX1*::*RUNX1T1*, *KMT2A* rearrangements, and *CBFB*::*MYH11*—suggesting a lower diversity of fusion events compared to other age groups.

In B-ALL, the overall pathogenic fusion detection rate was 59.3%, which aligns with previous studies [13]. However, the <15-year-old group, which is typically considered the most prevalent age group for B-ALL, showed a relatively low detection rate of 38.5%. Notably, *BCR*::*ABL1* was absent in the <15 year age group but increased in prevalence with age, reaching up to 60.0% of patients older than 60 years old.

*ZNF384* rearrangement was the second most frequent fusion in the overall B-ALL cohort and was the most common fusion in patients aged <15 years. It was also the second most frequent fusion in both the AYA and 40–60-year age groups, showing high proportions ranging from 10% to 18%. *ZNF384* is not included in several commonly used targeted panels such as Archer FusionPlex Pan-Heme Panel or HemaVision^®^ kit, suggesting underestimation of its prevalence. Previous studies reported the frequency of *ZNF384* rearrangement in B-ALL as ranging from 5 to 10%, with a higher prevalence in adults compared to children [24,26]. However, despite the limitation of a relatively small sample size, our study demonstrated a comparable frequency of approximately 10% in both pediatric and adult groups, suggesting that *ZNF384* rearrangement may not be uncommon in pediatric B-ALL in our East Asian cohort. Additionally, 9 cases with failed detection of *ZNF384* rearrangement by chromosome karyotyping highlight the necessity of whole RNA-seq. These discrepancies emphasize the need for further investigation using whole RNA-seq to better define the true prevalence and age distribution of *ZNF384* rearrangements.

Unlike targeted RNA sequencing, whole RNA-seq lacks consensus on the required sequencing output for confident fusion detection. Initially, some samples in our institution were sequenced to more than 1 billion reads (~100 GB per fastq file), but the large data volume and prolonged analysis times raised concerns, particularly when targetable fusions such as *BCR*::*ABL1* must be reported rapidly for clinical decision-making. While clinical reporting at our center has typically used data exceeding 400 million mapped reads, we performed systematic subsampling to determine whether reduced data size could ensure high sensitivity. Our results suggest that 100 million reads is sufficient to detect most fusions with medium or high confidence using Arriba, while 300 million reads is recommended for detection under high-confidence criteria only.

Most clinically relevant gene fusions exhibited split reads, allowing high-confidence detection in Arriba. However, two notable limitations existed. First, a few gene fusions, such as *ETV6*::*RUNX1* and *NUP98*::*NSD1*, demonstrated fewer split reads. Therefore, it is practical to understand the specific characteristics of gene fusions to avoid detection failure in hematologic malignancies. Several factors can affect the number of split reads and the detection of gene fusions, such as the depth of coverage, uniformity of coverage, RNA concentrations, and transcript length [27,28]. However, we were unable to pinpoint a precise cause. Furthermore, it is plausible that the gene fusion transcripts included in this study inadvertently included samples with gene fusions that inherently possess low transcript counts. Regarding *ETV6*::*RUNX1*, the results of a previous study did not indicate that this gene fusion is frequently associated with a relatively low transcript count in RNA-seq [29]. Meanwhile, the expression level of leukemic fusion transcripts is influenced by various factors such as promoter strength, transcript stability, and clonal composition, indicating the need for further investigation.

Second, prior to reaching the high sequencing output of 400 million reads, the rate of increase in mean split reads began to decline from approximately 200–300 million reads. Therefore, we remain skeptical about using such high sequencing output due to the reduced rate of increase and prolonged analysis time. We recommend a sequencing output of 300 million reads when analyzing only high-confidence calls and at least 100 million reads when including medium- or high-confidence calls.

While the optimal sequencing output can vary depending on the aim of the experiment and remains a topic of debate, some authors have suggested that up to 100 million reads are necessary for effective detection and quantification of gene fusions. Moreover, it has been recommended that more than 200 million paired-end reads are required to detect a full range of rare fusions [28,30]. As mentioned above, we recommend a sequencing output of 300 million reads when calling only high-confidence fusions and one of 100 million reads or more when medium- or high-confidence fusions are included. Meanwhile, including medium-confidence fusions increases the number of results requiring interpretation. Therefore, if the processing time does not significantly differ, extracting 300 million reads and analyzing only high-confidence calls can be an efficient alternative approach.

As clinical laboratory infrastructure continues to improve—with increasing sequencing capacity, faster processing speeds, and more accessible storage solutions—we believe that whole RNA-seq holds the potential to gradually replace DNA sequencing and conventional chromosomal karyotyping in certain diagnostic contexts. First, in both the WHO 5th classification and the 2022 ICC guidelines [5,6,7,8], gene fusions form the cornerstone of diagnostic definitions in most entities. Therefore, by enabling a more comprehensive search across a broad range of leukemic fusions compared to chromosomal karyotyping, RT-PCR, or targeted RNA-seq, whole RNA-seq can facilitate more accurate diagnosis and improved patient management. Second, whole RNA-seq also allows for the detection of additional genomic alterations, such as single nucleotide polymorphisms or copy number variations [31,32], further expanding its diagnostic utility. While DNA-based assays are traditionally used for variant detection, RNA provides a more functionally relevant measure by capturing expressed variants, which may better reflect downstream protein function and pathogenic potential [33]. Lastly, as demonstrated in this study, DNA sequencing inherently lacks the capacity to detect fusion transcripts, and conventional karyotyping—limited by the analysis of 20 to 40 metaphases—may miss low-frequency or cryptic rearrangements that whole RNA-seq can capture with higher sensitivity. These findings suggest that whole RNA-seq can serve as a powerful complementary platform alongside conventional cytogenetic and DNA-based assays in hematologic diagnostics, particularly for the detection of clinically relevant fusion events and cryptic rearrangements.

There were several limitations in this study. First, although Arriba is one of the premier tools in whole RNA-seq analysis, the results concerning gene fusions might vary by algorithm. Because biologically relevant read-through fusions such as *UBTF*::*ATXN7L3* may be removed by default filtering settings, careful interpretation and selective reanalysis strategies may be necessary in certain clinical contexts. Additional limitations of RNA-seq include challenges in detecting genomic rearrangements that do not produce functional fusion transcripts. In particular, rearrangements involving enhancer hijacking—such as those affecting *MECOM*, *IGH*, and *MYC*—may evade detection, as they are primarily characterized by aberrant gene expression rather than the formation of chimeric transcripts [34,35]. Second, the number of patients included in this study was insufficient to accurately assess the prevalence of gene fusions across different age groups and diagnostic categories. Third, as described above, it remained unclear why certain gene fusions demonstrated low split reads. Regardless of the underlying cause, it is crucial to interpret such findings with caution, particularly when clinically significant gene fusions are identified with low read support or confidence. In such cases, it may be beneficial to confirm the presence of these fusions using alternative methods. Future prospective studies using standardized sequencing pipelines and harmonized bioinformatic workflows will be necessary to validate the optimal sequencing thresholds identified in this study. In addition, further multicenter investigations may help clarify the prevalence and clinical significance of rare or newly recognized fusion events across different patient populations.

In this retrospective study, we demonstrated the clinical utility of whole RNA-seq for identifying pathogenic gene fusions across diverse subtypes of acute leukemia in a large real-world clinical setting. Our findings highlight the added diagnostic value of whole RNA-seq over conventional cytogenetic methods and targeted fusion panels. Notably, through systematic subsampling, we provide empirical evidence supporting a minimum sequencing output of 100 million reads for medium-confidence detection and 300 million reads for high-confidence detection in routine clinical practice. These results offer a practical framework for the implementation and standardization of fusion testing by whole RNA-seq in hematologic malignancies.

## Figures and Tables

**Table 1:** Demographic features of acute leukemia patients.

	AML	B-ALL	T-ALL	MPAL
*n*	205	81	10	5
Male sex (%)	111 (54.1)	35 (43.2)	5 (50.0)	4 (80.0)
Age (mean, range)	59.0 (2.6–90.0)	25.3 (0.6–82.2)	23.5 (5.2–69.8)	51.8 (28.5–79.9)
Pathogenic fusion detected, n (%)	60 (29.3)	48 (59.3)	2 (20.0)	3 (60.0)
Fusion of unknown significance detected, n (%)	15 (7.3)	1 (1.2)	1 (10.0)	1 (20.0)
Pathogenic or fusion of unknown significance detected, n (%)	75 (36.6)	49 (60.5)	3 (30.0)	4 (80.0)

Abbreviations, AML, acute myeloid leukemia; B-ALL, B-lymphoblastic leukemia; T-ALL, T-lymphoblastic leukemia; MPAL, mixed phenotype acute leukemia.

**Table 2:** Comparison of chromosome karyotyping and RNA sequencing.

Diagnosis	RNA Sequencing	Known Abnormality by Karyotyping	No Known Abnormality by Karyotyping	No Orthogonal Data Applicable
Total	Detected corresponding to karyotyping	73	0	0
	Detected, not corresponding to karyotyping	0	32	8
	Not detected	2	176	10
AML	Detected corresponding to karyotyping	50	0	0
	Detected, not corresponding to karyotyping	0	9	1
	Not detected	2	140	3
B-ALL	Detected corresponding to karyotyping	21	0	0
	Detected, with additional RNA-seq abnormality	1	0	0
	Detected, not corresponding to karyotyping	0	19	7
	Not detected	0	26	7
T-ALL	Detected corresponding to karyotyping	0	0	0
	Detected, not corresponding to karyotyping	0	2	0
	Not detected	0	8	0
MPAL	Detected corresponding to karyotyping	1	0	0
	Detected, not corresponding to karyotyping	0	2	0
	Not detected	0	2	0

Abbreviations: AML, acute myeloid leukemia; B-ALL, B-lymphoblastic leukemia; T-ALL, T-lymphoblastic leukemia; MPAL, mixed-phenotype acute leukemia.

**Table 3:** Detection rates for each condition, based on Arriba results (**A**) High confidence and (**B**) Medium/high confidence levels.

(**A**)												
**Type**	**3.125 M**	**6.25 M**	**12.5 M**	**25 M**	**50 M**	**100 M**	**150 M**	**200 M**	**250 M**	**300 M**	**350 M**	**400 M**
*BCR*::*ABL1 (b3a2)*	50% (2/4) [6.8–93.2%]	75% (3/4) [19.4–99.4%]	100% (4/4) [39.8–100.0%]	100% (4/4) [39.8–100.0%]	100% (4/4) [39.8–100.0%]	100% (4/4) [39.8–100.0%]	100% (4/4) [39.8–100.0%]	100% (4/4) [39.8–100.0%]	100% (4/4) [39.8–100.0%]	100% (4/4) [39.8–100.0%]	100% (4/4) [39.8–100.0%]	100% (4/4) [39.8–100.0%]
*BCR*::*ABL1 (e1a2)*	40% (4/10) [12.2–73.8%]	80% (8/10) [44.4–97.5%]	90% (9/10) [55.5–99.7%]	90% (9/10) [55.5–99.7%]	100% (10/10) [69.2–100.0%]	100% (10/10) [69.2–100.0%]	100% (10/10) [69.2–100.0%]	100% (10/10) [69.2–100.0%]	100% (10/10) [69.2–100.0%]	100% (10/10) [69.2–100.0%]	100% (10/10) [69.2–100.0%]	100% (10/10) [69.2–100.0%]
*CBFB*::*MYH11*	22% (2/9) [2.8–60.0%]	78% (7/9) [40.0–97.2%]	89% (8/9) [51.8–99.7%]	100% (9/9) [66.4–100.0%]	100% (9/9) [66.4–100.0%]	100% (9/9) [66.4–100.0%]	100% (9/9) [66.4–100.0%]	100% (9/9) [66.4–100.0%]	100% (9/9) [66.4–100.0%]	100% (9/9) [66.4–100.0%]	100% (9/9) [66.4–100.0%]	100% (9/9) [66.4–100.0%]
*EP300*::*ZNF384*	67% (2/3) [9.4–99.2%]	67% (2/3) [9.4–99.2%]	100% (3/3) [29.2–100.0%]	100% (3/3) [29.2–100.0%]	100% (3/3) [29.2–100.0%]	100% (3/3) [29.2–100.0%]	100% (3/3) [29.2–100.0%]	100% (3/3) [29.2–100.0%]	100% (3/3) [29.2–100.0%]	100% (3/3) [29.2–100.0%]	100% (3/3) [29.2–100.0%]	100% (3/3) [29.2–100.0%]
*ETV6*::*RUNX1*	0% (0/4) [0.0–60.2%]	0% (0/4) [0.0–60.2%]	0% (0/4) [0.0–60.2%]	0% (0/4) [0.0–60.2%]	50% (2/4) [6.8–93.2%]	50% (2/4) [6.8–93.2%]	50% (2/4) [6.8–93.2%]	75% (3/4) [19.4–99.4%]	75% (3/4) [19.4–99.4%]	100% (4/4) [39.8–100.0%]	100% (4/4) [39.8–100.0%]	100% (4/4) [39.8–100.0%]
*KMT2A*::*AFF1*	0% (0/4) [0.0–60.2%]	0% (0/4) [0.0–60.2%]	50% (2/4) [6.8–93.2%]	100% (4/4) [39.8–100.0%]	100% (4/4) [39.8–100.0%]	100% (4/4) [39.8–100.0%]	100% (4/4) [39.8–100.0%]	100% (4/4) [39.8–100.0%]	100% (4/4) [39.8–100.0%]	100% (4/4) [39.8–100.0%]	100% (4/4) [39.8–100.0%]	100% (4/4) [39.8–100.0%]
*KMT2A*::*MLLT3*	25% (1/4) [0.6–80.6%]	50% (2/4) [6.8–93.2%]	75% (3/4) [19.4–99.4%]	75% (3/4) [19.4–99.4%]	100% (4/4) [39.8–100.0%]	100% (4/4) [39.8–100.0%]	100% (4/4) [39.8–100.0%]	100% (4/4) [39.8–100.0%]	100% (4/4) [39.8–100.0%]	100% (4/4) [39.8–100.0%]	100% (4/4) [39.8–100.0%]	100% (4/4) [39.8–100.0%]
*PML*::*RARA*	18% (2/11) [2.3–51.8%]	45% (5/11) [16.7–76.6%]	91% (10/11) [58.7–99.8%]	100% (11/11) [71.5–100.0%]	100% (11/11) [71.5–100.0%]	100% (11/11) [71.5–100.0%]	100% (11/11) [71.5–100.0%]	100% (11/11) [71.5–100.0%]	100% (11/11) [71.5–100.0%]	100% (11/11) [71.5–100.0%]	100% (11/11) [71.5–100.0%]	100% (11/11) [71.5–100.0%]
*RUNX1*::*MECOM*	25% (1/4) [0.6–80.6%]	50% (2/4) [6.8–93.2%]	50% (2/4) [6.8–93.2%]	50% (2/4) [6.8–93.2%]	75% (3/4) [19.4–99.4%]	100% (4/4) [39.8–100.0%]	100% (4/4) [39.8–100.0%]	100% (4/4) [39.8–100.0%]	100% (4/4) [39.8–100.0%]	100% (4/4) [39.8–100.0%]	100% (4/4) [39.8–100.0%]	100% (4/4) [39.8–100.0%]
*RUNX1*::*RUNX1T1*	69% (9/13) [38.6–90.9%]	92% (12/13) [64.0–99.8%]	100% (13/13) [75.3–100.0%]	100% (13/13) [75.3–100.0%]	100% (13/13) [75.3–100.0%]	100% (13/13) [75.3–100.0%]	100% (13/13) [75.3–100.0%]	100% (13/13) [75.3–100.0%]	100% (13/13) [75.3–100.0%]	100% (13/13) [75.3–100.0%]	100% (13/13) [75.3–100.0%]	100% (13/13) [75.3–100.0%]
*TCF3*::*PBX1*	100% (5/5) [47.8–100.0%]	100% (5/5) [47.8–100.0%]	100% (5/5) [47.8–100.0%]	100% (5/5) [47.8–100.0%]	100% (5/5) [47.8–100.0%]	100% (5/5) [47.8–100.0%]	100% (5/5) [47.8–100.0%]	100% (5/5) [47.8–100.0%]	100% (5/5) [47.8–100.0%]	100% (5/5) [47.8–100.0%]	100% (5/5) [47.8–100.0%]	100% (5/5) [47.8–100.0%]
*TCF3*::*ZNF384*	25% (1/4) [0.6–80.6%]	50% (2/4) [6.8–93.2%]	75% (3/4) [19.4–99.4%]	100% (4/4) [39.8–100.0%]	100% (4/4) [39.8–100.0%]	100% (4/4) [39.8–100.0%]	100% (4/4) [39.8–100.0%]	100% (4/4) [39.8–100.0%]	100% (4/4) [39.8–100.0%]	100% (4/4) [39.8–100.0%]	100% (4/4) [39.8–100.0%]	100% (4/4) [39.8–100.0%]
Other variants *	8% (1/12) [0.2–38.5%]	17% (2/12) [2.1–48.4%]	50% (6/12) [21.1–78.9%]	58% (7/12) [27.7–84.8%]	75% (9/12) [42.8–94.5%]	92% (11/12) [61.5–99.8%]	100% (12/12) [73.5–100.0%]	100% (12/12) [73.5–100.0%]	100% (12/12) [73.5–100.0%]	100% (12/12) [73.5–100.0%]	100% (12/12) [73.5–100.0%]	100% (12/12) [73.5–100.0%]
Total	34% (30/87) [24.6–45.4%]	57% (50/87) [46.4–68.0%]	78% (68/87) [68.0–86.3%]	85% (74/87) [75.8–91.8%]	93% (81/87) [85.6–97.4%]	97% (84/87) [90.3–99.3%]	98% (85/87) [91.9–99.7%]	99% (86/87) [93.8–100.0%]	99% (86/87) [93.8–100.0%]	100% (87/87) [95.8–100.0%]	100% (87/87) [95.8–100.0%]	100% (87/87) [95.8–100.0%]
(**B**)												
**Type**	**3.125 M**	**6.25 M**	**12.5 M**	**25 M**	**50 M**	**100 M**	**150 M**	**200 M**	**250 M**	**300 M**	**350 M**	**400 M**
*BCR*::*ABL1 (b3a2)*	50% (2/4) [6.8–93.2%]	75% (3/4) [19.4–99.4%]	100% (4/4) [39.8–100.0%]	100% (4/4) [39.8–100.0%]	100% (4/4) [39.8–100.0%]	100% (4/4) [39.8–100.0%]	100% (4/4) [39.8–100.0%]	100% (4/4) [39.8–100.0%]	100% (4/4) [39.8–100.0%]	100% (4/4) [39.8–100.0%]	100% (4/4) [39.8–100.0%]	100% (4/4) [39.8–100.0%]
*BCR*::*ABL1 (e1a2)*	90% (9/10) [55.5–99.7%]	100% (10/10) [69.2–100.0%]	100% (10/10) [69.2–100.0%]	100% (10/10) [69.2–100.0%]	100% (10/10) [69.2–100.0%]	100% (10/10) [69.2–100.0%]	100% (10/10) [69.2–100.0%]	100% (10/10) [69.2–100.0%]	100% (10/10) [69.2–100.0%]	100% (10/10) [69.2–100.0%]	100% (10/10) [69.2–100.0%]	100% (10/10) [69.2–100.0%]
*CBFB*::*MYH11*	44% (4/9) [13.7–78.8%]	89% (8/9) [51.8–99.7%]	89% (8/9) [51.8–99.7%]	100% (9/9) [66.4–100.0%]	100% (9/9) [66.4–100.0%]	100% (9/9) [66.4–100.0%]	100% (9/9) [66.4–100.0%]	100% (9/9) [66.4–100.0%]	100% (9/9) [66.4–100.0%]	100% (9/9) [66.4–100.0%]	100% (9/9) [66.4–100.0%]	100% (9/9) [66.4–100.0%]
*EP300*::*ZNF384*	67% (2/3) [9.4–99.2%]	67% (2/3) [9.4–99.2%]	100% (3/3) [29.2–100.0%]	100% (3/3) [29.2–100.0%]	100% (3/3) [29.2–100.0%]	100% (3/3) [29.2–100.0%]	100% (3/3) [29.2–100.0%]	100% (3/3) [29.2–100.0%]	100% (3/3) [29.2–100.0%]	100% (3/3) [29.2–100.0%]	100% (3/3) [29.2–100.0%]	100% (3/3) [29.2–100.0%]
*ETV6*::*RUNX1*	25% (1/4) [0.6–80.6%]	25% (1/4) [0.6–80.6%]	50% (2/4) [6.8–93.2%]	75% (3/4) [19.4–99.4%]	75% (3/4) [19.4–99.4%]	100% (4/4) [39.8–100.0%]	100% (4/4) [39.8–100.0%]	100% (4/4) [39.8–100.0%]	100% (4/4) [39.8–100.0%]	100% (4/4) [39.8–100.0%]	100% (4/4) [39.8–100.0%]	100% (4/4) [39.8–100.0%]
*KMT2A*::*AFF1*	25% (1/4) [0.6–80.6%]	25% (1/4) [0.6–80.6%]	75% (3/4) [19.4–99.4%]	100% (4/4) [39.8–100.0%]	100% (4/4) [39.8–100.0%]	100% (4/4) [39.8–100.0%]	100% (4/4) [39.8–100.0%]	100% (4/4) [39.8–100.0%]	100% (4/4) [39.8–100.0%]	100% (4/4) [39.8–100.0%]	100% (4/4) [39.8–100.0%]	100% (4/4) [39.8–100.0%]
*KMT2A*::*MLLT3*	50% (2/4) [6.8–93.2%]	75% (3/4) [19.4–99.4%]	75% (3/4) [19.4–99.4%]	75% (3/4) [19.4–99.4%]	100% (4/4) [39.8–100.0%]	100% (4/4) [39.8–100.0%]	100% (4/4) [39.8–100.0%]	100% (4/4) [39.8–100.0%]	100% (4/4) [39.8–100.0%]	100% (4/4) [39.8–100.0%]	100% (4/4) [39.8–100.0%]	100% (4/4) [39.8–100.0%]
*PML*::*RARA*	18% (2/11) [2.3–51.8%]	55% (6/11) [23.4–83.3%]	100% (11/11) [71.5–100.0%]	100% (11/11) [71.5–100.0%]	100% (11/11) [71.5–100.0%]	100% (11/11) [71.5–100.0%]	100% (11/11) [71.5–100.0%]	100% (11/11) [71.5–100.0%]	100% (11/11) [71.5–100.0%]	100% (11/11) [71.5–100.0%]	100% (11/11) [71.5–100.0%]	100% (11/11) [71.5–100.0%]
*RUNX1*::*MECOM*	50% (2/4) [6.8–93.2%]	50% (2/4) [6.8–93.2%]	75% (3/4) [19.4–99.4%]	75% (3/4) [19.4–99.4%]	75% (3/4) [19.4–99.4%]	100% (4/4) [39.8–100.0%]	100% (4/4) [39.8–100.0%]	100% (4/4) [39.8–100.0%]	100% (4/4) [39.8–100.0%]	100% (4/4) [39.8–100.0%]	100% (4/4) [39.8–100.0%]	100% (4/4) [39.8–100.0%]
*RUNX1*::*RUNX1T1*	100% (13/13) [75.3–100.0%]	100% (13/13) [75.3–100.0%]	100% (13/13) [75.3–100.0%]	100% (13/13) [75.3–100.0%]	100% (13/13) [75.3–100.0%]	100% (13/13) [75.3–100.0%]	100% (13/13) [75.3–100.0%]	100% (13/13) [75.3–100.0%]	100% (13/13) [75.3–100.0%]	100% (13/13) [75.3–100.0%]	100% (13/13) [75.3–100.0%]	100% (13/13) [75.3–100.0%]
*TCF3*::*PBX1*	100% (5/5) [47.8–100.0%]	100% (5/5) [47.8–100.0%]	100% (5/5) [47.8–100.0%]	100% (5/5) [47.8–100.0%]	100% (5/5) [47.8–100.0%]	100% (5/5) [47.8–100.0%]	100% (5/5) [47.8–100.0%]	100% (5/5) [47.8–100.0%]	100% (5/5) [47.8–100.0%]	100% (5/5) [47.8–100.0%]	100% (5/5) [47.8–100.0%]	100% (5/5) [47.8–100.0%]
*TCF3*::*ZNF384*	50% (2/4) [6.8–93.2%]	50% (2/4) [6.8–93.2%]	75% (3/4) [19.4–99.4%]	100% (4/4) [39.8–100.0%]	100% (4/4) [39.8–100.0%]	100% (4/4) [39.8–100.0%]	100% (4/4) [39.8–100.0%]	100% (4/4) [39.8–100.0%]	100% (4/4) [39.8–100.0%]	100% (4/4) [39.8–100.0%]	100% (4/4) [39.8–100.0%]	100% (4/4) [39.8–100.0%]
Other variants *	33% (4/12) [9.9–65.1%]	42% (5/12) [15.2–72.3%]	58% (7/12) [27.7–84.8%]	83% (10/12) [51.6–97.9%]	92% (11/12) [61.5–99.8%]	100% (12/12) [73.5–100.0%]	100% (12/12) [73.5–100.0%]	100% (12/12) [73.5–100.0%]	100% (12/12) [73.5–100.0%]	100% (12/12) [73.5–100.0%]	100% (12/12) [73.5–100.0%]	100% (12/12) [73.5–100.0%]
Total	56% (49/87) [45.3–66.9%]	70% (61/87) [59.4–79.5%]	86% (75/87) [77.1–92.7%]	94% (82/87) [87.1–98.1%]	97% (84/87) [90.3–99.3%]	100% (87/87) [95.8–100.0%]	100% (87/87) [95.8–100.0%]	100% (87/87) [95.8–100.0%]	100% (87/87) [95.8–100.0%]	100% (87/87) [95.8–100.0%]	100% (87/87) [95.8–100.0%]	100% (87/87) [95.8–100.0%]

* Other variants include *EBF1*::*PDGFRB*, *KMT2A*::*AFDN*, *KMT2A*::*ELL*, *NUP214*::*ABL1*, *NUP98*::*NSD1*, *UBTF*::*ATXN7L3*.

## Data Availability

The datasets used and/or analyzed during the current study are available from the corresponding author (Shin S) on reasonable request. The data are not publicly available due to privacy or ethical restrictions.

## Supplementary Materials

The following supporting information can be downloaded at https://www.mdpi.com/article/10.3390/cells15121048/s1, Figure S1: FASTQ-level validation set: mean split reads saturation curves; Table S1: AML cases with cytogenetic abnormalities not detected by whole RNA-seq and their possible biological explanations; Table S2: Detection rates for individual fusion variants across raw FASTQ-level downsampling depths, based on Arriba results (A) High confidence and (B) Medium/high confidence levels; Table S3: Average frequency of false-positive fusion calls per sample across downsampled sequencing depths.

## Abbreviations

The following abbreviations are used in this manuscript:

WHO	World Health Organization
NGS	Next-generation sequencing
RNA-seq	RNA sequencing
IRB	Institutional Review Board
AML	Acute myeloid leukemia
B-ALL	B-lymphoblastic leukemia
T-ALL	T-lymphoblastic leukemia
MPAL	Mixed phenotype acute leukemia
AYA	Adolescent and young adult
FISH	Fluorescence in situ hybridization
RT-PCR	Reverse transcriptase polymerase chain reaction
HSCT	Hematopoietic stem cell transplantation
ICC	International Consensus Classification
