# Supplementary figures and images for: Clinical Utility of Whole RNA Sequencing for Fusion Detection in Acute Leukemia
Source: Cells. 2026 Jun 8;15(12):1048. doi: 10.3390/cells15121048 (PMC13297425; doi:10.3390/cells15121048)

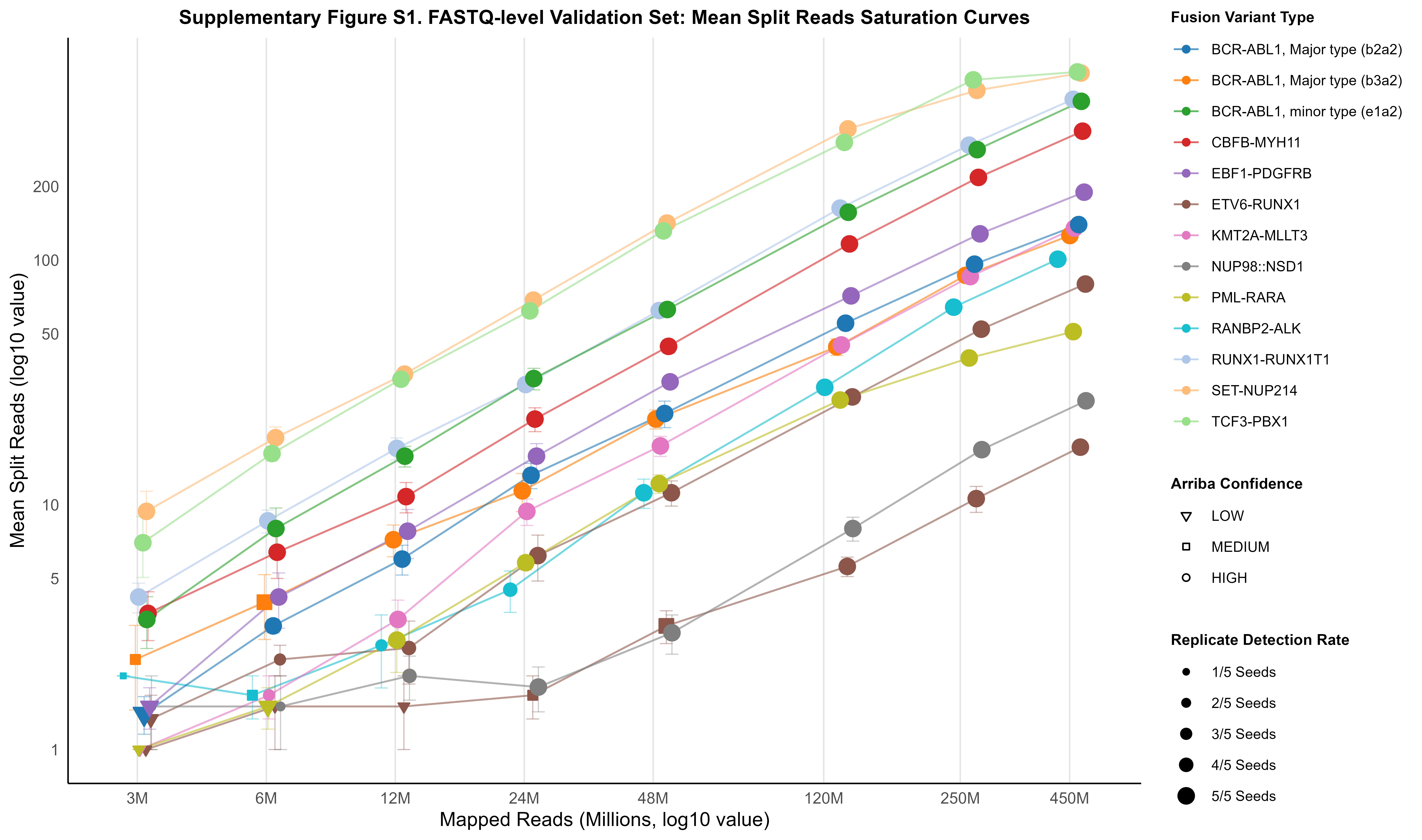

Supplement: Supplementary file 1 [file cells-15-01048-s001.zip › Supplementary_Figure_S1.png]
